# Immunohistochemical and ultrastructural properties of the larval ciliary band-associated strand in the sea urchin *Hemicentrotus pulcherrimus*

**DOI:** 10.1186/s12983-016-0159-8

**Published:** 2016-06-16

**Authors:** Hideki Katow, Tomoko Katow, Hiromi Yoshida, Masato Kiyomoto, Isao Uemura

**Affiliations:** Research Center for Marine Biology, Tohoku University, Asamushi, Aomori, Aomori 039-3501 Japan; Center of Research Instruments, Institute of Development, Aging and Cancer, Tohoku University, Sendai, 980-8575 Japan; Marine and Coastal Research Center, Ochanomizu University, Tateyama, Chiba 294-0301 Japan; Division of Biological Sciences, Tokyo Metropolitan University, Hachioji, Tokyo 192-0397 Japan

**Keywords:** GAD, 5HThpr, Encephalopsin, Synaptophysin, Adherens junctions, Epith-2, Sea urchin pluteus

## Abstract

**Background:**

The swimming activity of sea urchin larvae is dependent on the ciliary band (CB) on the larval surface and is regulated by several neurotransmitters, including serotonin (5HT), dopamine, and γ-aminobutyric acid (GABA). However, the CB signal transmission mechanism remains unknown. The present study investigated the structural relationship between the CB and external signal receptors by immunohistochemical and transmission electron microscopic analyses of sea urchin, *Hemicentrotus pulcherrimus*, larvae.

**Results:**

Glutamate decarboxylase (GAD; GABA synthetase) was detected in a strand of multiple cells along the circumoral CB in 6-arm plutei. The GAD-expressing strand was closely associated with the CB on the oral ectoderm side. The ciliary band-associated strand (CBAS) also expressed the 5HT receptor (5HThpr) and encephalopsin (ECPN) throughout the cytoplasm and comprised 1- to 2-μm diameter axon-like long stretched regions and sporadic 6- to 7-μm diameter bulbous nucleated regions (perikarya) that protruded into the oral ectoderm side. Besides the laterally polarized morphology of the CBAS cells, Epith-2, which is the epithelial lateral cell surface-specific protein of the sea urchin embryo and larva, was expressed exclusively by perikarya but not by the axon-like regions. The CBAS exposed its narrow apical surface on the larval epithelium between the CB and squamous cells and formed adherens junctions (AJs) on the apical side between them. Despite the presence of the CBAS axon-like regions, tubulins, such as α-, β-, and acetylated α-tubulins, were not detected. However, the neuroendocrine cell marker protein synaptophysin was detected in the axon-like regions and in bouton-like protrusions that contained numerous small ultrastructural vesicles.

**Conclusions:**

The unique morphology of the CBAS in the sea urchin larva epithelium had not been reported. The CBAS expresses a remarkable number of receptors to environmental stimuli and proteins that are probably involved in signal transmission to the CB. The properties of the CBAS explain previous reports that larval swimming is triggered by environmental stimuli and suggest crosstalk among receptors and potential plural sensory functions of the CBAS.

## Background

The biphasic life cycle of marine invertebrates is characterized by metamorphosis controlled by environmental and internal stimuli including neurotransmitters [1–4]. Sea urchin larval metamorphosis is triggered by γ-aminobutyric acid (GABA) derived from red algae in the larval environment [1, 5, 6], suggesting the involvement of a GABA receptor during metamorphosis that also includes swimming activity [7]. Sea urchin larvae also respond to other neurotransmitters, such as serotonin (5HT; [8, 9]) and dopamine (DA; [10]) in addition to light [11]. All of these stimuli are available in the sea urchin habitat, such as 5HT from red algae [12] and DA from green algae [13, 14].

The major driving force for sea urchin larval swimming is generated by beating of cilia on the ciliary band (CB) of larval arms [15], which is regulated by DA [10, 16], 5HT [8, 9, 16–18], and GABA [7]. 5HT initiates the cytoplasmic calcium ion-releasing signal to the ciliated epithelial cells through the coelomic network of 5HT receptor (5HThpr)-expressing cells [8, 9].

The DRD1 DA receptor [10] and a GABA-ergic signaling system, which includes the GABA_A_ receptor (GABA_A_R) and GABA_A_R-associated protein, has been localized along the CB [7]. A strand of glutamate decarboxylase (GAD)-expressing cells (GADCs) along the CB has also been reported in plutei [7]. The CB-associated strand (CBAS) of GADCs appears at about the 2-arm pluteus stage and encircles the oral ectoderm by the 6-arm pluteus stage [7]. These previous reports suggest that the CBAS functions as a GABA sensory organ. Larval swimming is regulated by the photoreceptor protein encephalopsin (ECPN). However, ECPN is expressed in a subgroup of blastocoelar cells along with 5HT receptor (5HThpr) [8], despite that these cells are not closely localized with the CB [11].

The present study (1) clarified the morphological details of the CBAS, (2) located 5HThpr and ECPN near the CB using whole-mount immunohistochemistry (WMIHC) with combinations of antibodies and transmission electron microscopy (TEM), and (3) hypothesized the architecture of potential crosstalk among these receptors of environmental stimuli at or near the CBAS.

## Results

### The CBAS is located on the oral ectoderm side along the CB

**Fig. 1:**
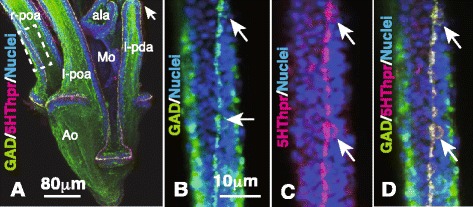
The multicellular strand of the larval arms co-expresses glutamate decarboxylase (GAD) and the 5HT receptor (5HThpr). **a** The oral ectoderm and the aboral ectoderm are bordered by a multicellular strand that expresses GAD (*green*) and 5HThpr (*red*) in the 6-arm pluteus. ala, anterolateral arms; arrow, tip of arm; l-pda, left posterodorsal arm; l-, r-poa, left- and right-postoral arms; Mo, mouth. **b** Fifteen μm thick optical section of the part of the arm indicated by the dotted box in (**a**) shows GAD and nuclei. Apparent smeared green on the larval surface reflects blastocoelar GAD cells. **c** Same area as (**b**) shows 5HThpr and nuclei. **d** Merged image between (**b**) and (**c**) shows GAD and 5HThpr expression in the same place as (**b**, **c**). Arrows, perikarya regions

**Fig. 2:**
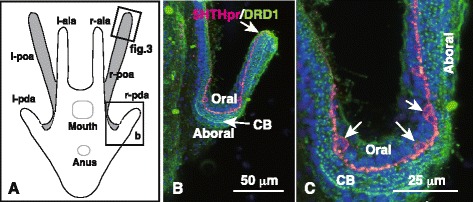
The ciliary band-associated strand (CBAS) resides on the oral ectoderm side of the ciliary band. (**a**) Schematic aboral view of 6-arm pluteus of (B). Gray area, oral ectoderm; l- and r-ala, left- and right-anterolateral arms; l- and r-pda, left- and right-posterodorsal arms; l- and r-poa, left and right-postoral arms. (**b**) Confocal laser scanning microscopic image of triple-stained whole-mount immunohistochemistry of the r-pda indicated by box (b) in (A) shows the 5HT receptor (5HThpr)-expressing CBAS (*red*) on the oral ectoderm side (Oral) of the dopamine receptor (DRD1)-expressing ciliary band (CB; *green dots*). Nuclei were stained with 4′,6-diamidino-2-phenylindole (*blue*; DAPI). (**c**) High-magnification image of the CB of the r-pda indicated in (B) shows characteristic multiple lines of DRD1-positive basal bodies of the cilia. Perikarya (*arrows*) protrude into the oral ectoderm side (Oral). Aboral, aboral ectoderm area

**Fig. 3:**
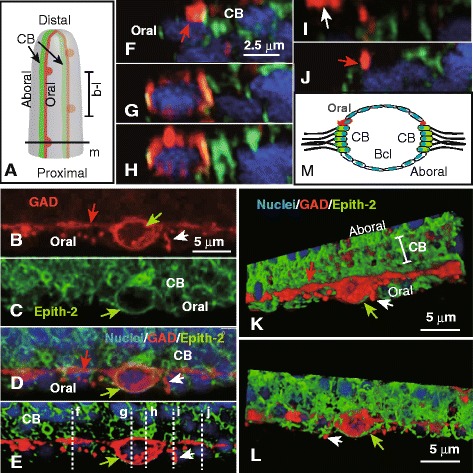
Immunohistochemical cell surface property of the ciliary band-associated strand (CBAS). (**a**) Schematic of arm tip region indicated by box (Fig. 3) in Fig. 2a. Red line, CBAS with perikarya (*red dots*). Vertical bar (b–l), approximate region of image (b–l). (**b**) Glutamate decarboxylase (GAD) expression in the CBAS. (**c**) Epith-2 expression in the perikaryon (*green arrow*). (**d**) Merged image between (b) and (c). (**e**) Hybrid Super Resolution software (HSR)-processed image of (d) shows locations of the optical cross-sections (*dotted lines*). (**f**–**j**) Optical cross-sections indicated by vertical dotted lines (f–j) in (e). (**k**) HSR-processed 3D reconstructed image of the CBAS. (**l**) The other side of image (k) after rotation mostly covered by the Epith-2 signal. (**m**) Schematic of cross-section of arm indicated by bar (m) in (a). Bcl, blastocoel. Nuclei in (d–l) were stained with 4′,6-diamidino-2-phenylindole (blue). Aboral, aboral ectoderm regiuon; CB, ciliary band area; green arrow, perikaryon; Oral, oral ectoderm region; red arrow, axon-like region; white arrow, apical protrusion

The CBAS appeared during the 2–4-arm pluteus stage and further extended into all larval arms in 6-arm plutei (Fig. 1a). Because the CB formed at the border between the oral ectoderm and aboral ectoderm (Fig. 1a, b, d), Hp-DRD1 was chosen as a CB molecular marker to specify the exact location of the CBAS to the CB. DRD1 constitutes the basal body of the CB cilia [10]. However, the available anti-Hp-DRD1 polyclonal antibody (pAb) was raised in rabbits. To avoid using Abs raised in the same animal species, we changed the CBAS marker to a mouse anti-5HThpr pAb for use with the rabbit anti-GAD pAb (Fig. 1c). As our previous observations detected GAD expression in the same 5HThpr-expresing blastocoelar cells [19], we predicted that the GAD-expressing CBAS would also express 5HThpr. Expectedly, the present anti-5HThpr pAb detected binding to the CBAS (Fig. 1), which was observed as a strand of approximately 1.8 μm in diameter and was associated with periodical bulges of the 6- to 7-μm diameter nuclear regions (perikarya; arrows in Figs. 1b–d and 2c) exclusively on the oral ectoderm side along the DRD1-expressing CB (Fig. 2). The CB and the CBAS rounded the tip of the larval arm to the other side and encircled the oral ectoderm (arrows in Figs. 1a, 2b and 3a).

The epithelial cells of sea urchin embryos and larvae express the cell surface-specific Epith-2 protein on the lateral cell surface [20, 21], whereas initial GADCs appeared in the embryonic blastocoel through the epithelial-to-mesenchymal transition (EMT) from the ectoderm by losing all cell surface Epith-2 protein during early embryogenesis before CBAS formation [19, 22]. To examine if the CBAS re-expresses Epith-2 after its formation on the larval surface, larval postoral arms of 6-arm plutei were examined by triple-staining with Abs raised against GAD, Epith-2, and 4′,6-diamidino-2-phenylindole (DAPI) to stain nuclei. WMIHC revealed that the entire CBAS was positive for the anti-GAD pAb (Fig. 3b), whereas the mouse anti-Epith-2 monoclonal Ab (mAb) bound exclusively to the perikaryon surface, but not to the axon-like region (Fig. 3c, d) indicating the heterogeneous cell surface property of the CBAS.

Further analysis of the heterogeneous cell surface property of the CBAS was conducted by applying three-dimensional (3D) imaging using Amira 3D image processing software (FEI Visualization Sciences Group, Burlington, MA, USA). The 3D image was reconstructed using 75 slices of 250-nm-thick optical sections and was further processed using Hybrid Super Resolution software (HSR; Leica Microsystems Co., Tokyo, Japan) to obtain more detailed optical images (Fig. 3e). The Epith-2 protein in the perikaryon was not detected in the axon-like regions (Fig. 3e, f, i, j). The CBAS appeared to be located slightly below the apical surface of the larval body (Figs. 3f–l). The diagonal apical view of the CBAS revealed protrusions toward the larval apical surface (White arrow in Fig. 3b, d, e, i, k–l). These protrusions were approximately 0.4 μm in diameter, and one that extended near the perikaryon was approximately 3 μm long (Fig. 3I).

### CBAS ultrastructure

WMIHC of the larval arms localized the CBAS on the oral ectoderm side of the CB (Figs. 2 and 3) and detected an axon-like region of the CBAS as a circular cross-section. (Fig. 3f, j). However, because of the absence of an appropriate epithelial cell cytoplasm-specific molecular marker, the exact location of the CBAS in the epithelium, including whether the entire CBAS was exposed on the apical surface of the larvae or was just a part or was entirely covered by epithelium was not determined by the present WMIHC. Thus, further details of the CBAS were examined by TEM.

**Fig. 4:**
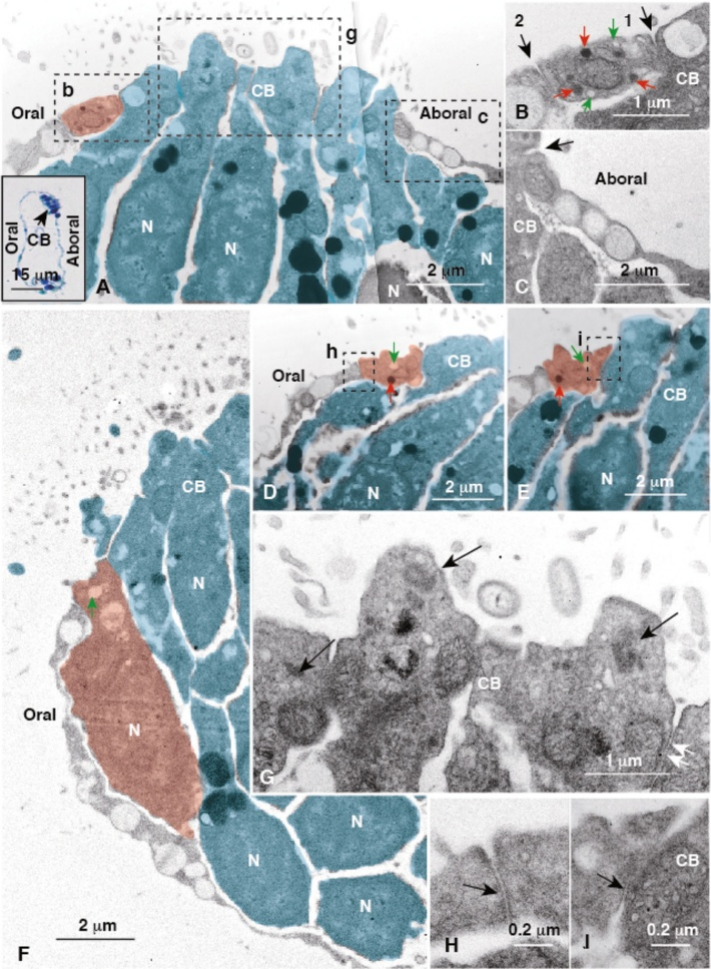
Transmission electron micrograph of the ciliary band (CB) of 6-arm pluteus. (**a**) Columnar CB cells (*blue*) adjacent to squamous cells on the aboral side (Aboral) and the rectangular cell (*red*) on the oral side (Oral). Inset, toluidine blue-stained thick cross-section of the arm shown by (a–e). (**b**) High-magnification of the region indicated by box (b) in (a). The rectangular cell possesses adherens junctions (AJ) between a CB cell (*arrow 1*) and a squamous cell (*arrow 2*). (**c**) High-magnification of the region indicated by box (c) in (a). Squamous cell adjacent to the CB cell (*arrow*). (**d**) Cross-section of the rectangular cell (*red*) adjacent to the CB cell (*blue*). (**e**) Cross-section of the rectangular cell (*red*) adjacent to the CB cell (*blue*) 40 μm away from the image in (d). (**f**) Cross-section of the perikaryon of the rectangular cell (*red*) adjacent to CB cells (*blue*). (**g**) High-magnification of the apical cytoplasm of the CB cells indicated by box (g) in (a). Arrows, basal bodies; double-arrow, AJ. (**h**) High-magnification of an AJ (*arrow*) between the rectangular cell and adjacent squamous cell shown by box (h) in (d). (**i**) High-magnification of an AJ (*arrow*) between the rectangular cell and the CB cell indicated by box (i) in (e). Aboral, the aboral ectoderm region; green arrows in (b, d-f), electron-translucent vesicles; N, nucleus in (a), (d–f); Oral, the oral ectoderm region, red arrows in (b, d, e), electron-dense vesicles

Before the TEM analysis, the oral-aboral orientation of the samples in transparent resin blocks was carefully examined under a microscope. According to a thick cross section analysis conducted before the TEM examination, the CB was a pair of symmetrical thick epithelial regions on both sides of an arm (Fig. 4a, inset). TEM showed that these thick epithelial regions comprised columnar ciliated cells (Fig. 4a, blue) that were adjacent to the thin, squamous areas on the oral and aboral sides (Fig. 4a–c). However, a further detailed examination showed that the oral ectoderm contained an approximate 0.5 × 0.8 μm sized cell in a rectangular cross-section on the oral ectoderm side of the CB (rectangular cells; Fig. 4a, red, b) that was adjacent to squamous cells on the far oral ectoderm region (Fig. 4a, b), whereas the aboral ectoderm comprised squamous cells without rectangular cells (Fig. 4a, c).

This initial TEM examination of rectangular cells suggested that they may be the CBAS. A pair of serial sections was made for further examination that was separated 40 μm from each other. In the first section (Fig. 4d) and the last section (Fig. 4e), a rectangular cell with similar dimensions to those of the cells in Fig. 4a, b was detected in a similar location adjacent to the CB. The distance between the two serial cross-sections encompassed 12–14 CB cells, indicating that the rectangular cells were part of a long strand of cells that stretched along the CB and resembled the axon-like region of the CBAS detected by WMIHC (Figs. 2 and 3). Another cross-section showed a significantly larger, approximately 6 × 2 μm, sized nucleated cell between the oral squamous cell and the CB (Fig. 4f, red) that resembled the perikaryon seen in the previous WMIHC (Figs. 2c and 3). The slight difference in the morphological dimensions of the CBAS determined by WMIHC (1.8 μm in diameter) and TEM (1.5 × 0.8 μm in diameter) was attributed to shrinkage of specimens during TEM preparation.

The CB cells protruded conically to the apical surface where the basal bodies were contained (Fig. 4g, arrows). Adjacent ciliary cells formed adherens junctions (AJs) on the most apical side of the lateral surface (Fig. 4g, double-arrow). Thus, the CBAS-like epithelial strand constituted part of the epithelium on the oral ectoderm side of the CB with AJs separated from adjacent cell surfaces by approximately 24 nm (Fig. 4h, i). The cytoplasm of the CBAS-like cells characteristically contained small 100 ± 20 nm (*n* = 8) diameter electron-dense and -translucent vacuoles (Fig. 4b, d–f).

**Fig. 5:**
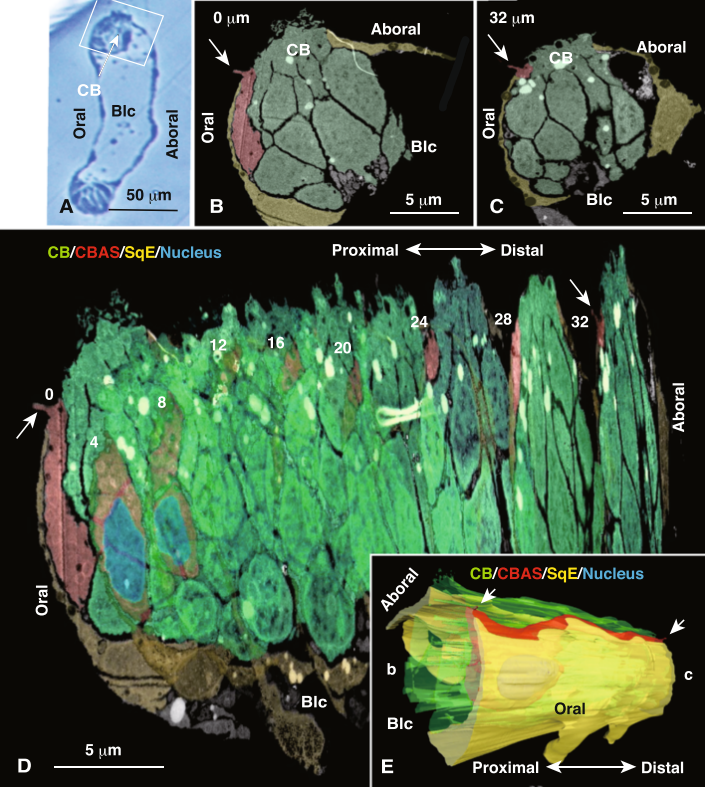
Three-dimensional (3D) tomographic reconstruction of the ciliary band (CB)-associated strand (CBAS) of the postoral arm. (**a**) Toluidine blue-stained thick cross-section of a larval arm of a 6-arm pluteus. Transmission electron microscopy (TEM, B–E) was applied to the region indicated by box. The larval arm was flattened during sample preparation. (**b**) TEM image of the first proximal section (0 μm) at the area indicated by box in (a). (**c**) TEM image of the last distal section 32 μm from the first section (B). (**d**) Stacked image of nine semi-serial cross-sections (0–32) by the Amira image processor. The CBAS (red) constitutes the initial bulbous proximal sections (0–8) to the long and thin stretch of strand towards the proximal sections (12–32). Squamous epithelium (SqE; *yellow*) encapsulates the major surface of the larval arm except the apical surface of the CB and the narrow strip of CBAS (*red*). (**e**) Smoothened 3D topographic reconstruction of (d). (b) and (c), proximal end indicated by (B) and distal end indicated by (c). Aboral, aboral ectoderm region; arrows, the apical protrusions; Blc, blastocoel; Oral, oral ectoderm region

Further analysis of serial thin sections was performed using the same larval postoral arm examined above to ensure morphological similarity of the ultrastructural CBAS-like structure to the immunohistochemical CBAS. The thin sections encompassed a 32 μm region towards the proximal region from the distal region (Fig. 5a–c). Nine 110 nm thick thin sections were made every 4 μm from the initial proximal section and were aligned to reconstruct a 3D image using Amira image software (Fig. 5d). The 3D reconstructed image showed the bulbous nucleated images in the initial three serial sections [Fig. 5d (0–8)] and the strand of rectangular cell images from the fourth section at 12 μm [Fig. 5d, (12)] to the last section at 32 μm [Fig. 5d (32)]. The image was smoothed by 3D topographical reconstruction processing using the Amira image processor (Fig. 5e) and indicated that the apical surface of the strand of rectangular cells and the bulbous regions constituted the CBAS-like apical strand throughout the entire length of the part of the larval arm examined in this study (Fig. 5e, red). A part of the apical surface of the CBAS-like structure was exposed on the apical surface of the larval arm at the region between the CB (Fig. 5e, green) and the oral squamous cells (Fig. 5e, yellow). Thus, the 3D reconstructed TEM image of the CBAS-like strand possessed consistent morphology to that of the CBAS obtained by WMIHC. This indicates that the CBAS has direct access to the larval environment by exposing part of the apical cell surface on the larval epithelium.

**Fig. 6:**
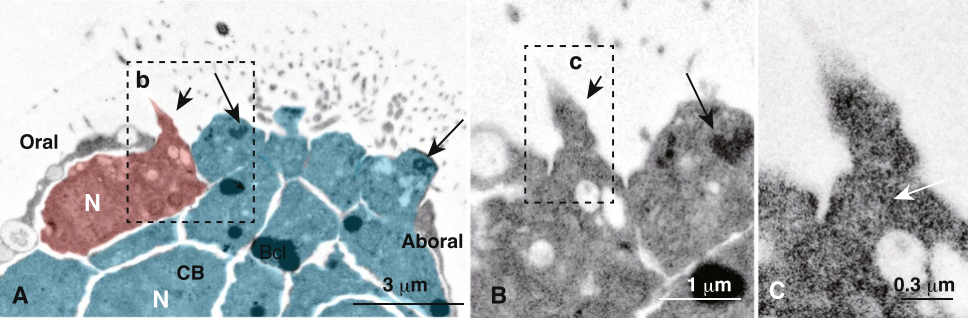
Transmission electron micrograph of the apical protrusion of the ciliary band-associated strand (CBAS, red). (**a**) The apical protrusion from the CBAS perikaryon was approximately 0.4 μm in diameter (*short arrow*). Its tip was obliquely decapitated approximately 1.8 μm from the base in this particular thin section. N, nucleus. (**b**) High-magnification of box (b) in (A). (**c**) High-magnification of box (c) in (b). Aboral, aboral ectoderm region; CB, the ciliary band (CB) cells; long arrows, basal bodies in the CB cells [blue in (a)]; Oral, oral ectoderm region; white arrow, small vesicles

Sporadic apical protrusions of approximately 0.4 μm in diameter were detected near the perikaryon and the axon-like region (Fig. 5b–e, arrows). The approximate diameter of a protrusion was similar to that detected by WMIHC (Fig. 3b, d, e, i, k–l). The length of a protrusion was approximately 3 μm (Fig. 3b). The base of the apical protrusion was not associated with the basal body, and no microtubules were seen in the stem of the protrusion (Fig. 6a, b). Instead, its cytoplasm contained numerous 40–80 nm diameter vesicles (Fig. 6c).

### Encephalopsin expression in the CBAS

**Fig. 7:**
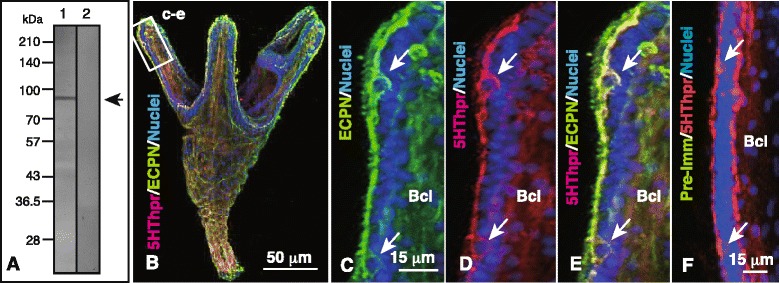
Immunochemical detection of encephalopsin (ECPN) in the ciliary band-associated strand (CBAS). (**a**) Immunoblotting of the 4-arm plutei lysate. Lane 1, rabbit anti-Hp-ECPN pAb; Lane 2, rabbit pre-immune serum. Arrow, 76 kDa region. (b–) Confocal laser scanning micrographs of whole-mount immunohistochemistry of 4-arm pluteus. Nuclei were stained with 4′,6-diamidino-2-phenylindole (DAPI, *blue*). (**b**) Triple-stained aboral view of 4-arm pluteus. (c–e) High-magnification image of a postoral arm indicated by box (c–e) in (B). (**c**) ECPN (*green*) at the CBAS. (**d**) 5HThpr (*red*) at the CBAS. (**e**) Merged image between (c) and (d). (**f**) Arm of a 6-arm pluteus triple-stained with rabbit pre-immune serum (*green*, negative), mouse anti-5HThpr (*red*), and DAPI. Arrows in (c–f), perikarya of the CBAS; Bcl, blastocoel

WMIHC detected simultaneous GAD and 5HThpr co-expression in the CBAS (Fig. 1), indicating that the CBAS retains a similar protein expression pattern to that of blastocoelar GADCs [19]. The blastocoelar cells in the gastrula stage include ECPN-expressing cells (ECPN cells) involved in photosensitive larval vertical migration [11]. The present anti-Hp-ECPN pAb was raised in rabbits, and it immunospecifically bound to a single band in the 76 kDa region by immunoblotting (Fig. 7a), which was identical to that of our previous study conducted using a mouse anti-Hp-ECPN pAb [11]. It is unknown whether there is any relationship between these ECPN cells and the present CBAS.

The present study detected ECPN expression in the CBAS by WMIHC using 4-arm plutei (Fig. 7b, c). The present triple-stain with anti-5HThpr pAb and DAPI detected an ECPN-expressing site in the same area of the CBAS (Fig. 7b, d, e). The negative control test using rabbit pre-immune serum did not detect any positivity in the larval arm (Fig. 7f). Thus, the CBAS retained the protein expression pattern of the blastocoelar GADCs and ECPN cells. The present triple-staining also detected an ECPN and 5HThpr-co-expressing blastocoelar network (Fig. 7c–e, Bcl) as we reported previously [11].

### Synaptophysin (Syn) expression in the CBAS

Together with our previous reports that larval swimming activity is regulated by serotonergic [8, 9, 18, 23], GABAergic [7], and photoreceptor signal transmission systems [11] and the present results that co-expression of the proteins responsible for these systems in the CBAS suggest that the CBAS possesses signal transmission proteins, such as Syn [24, 25].

**Fig. 8:**
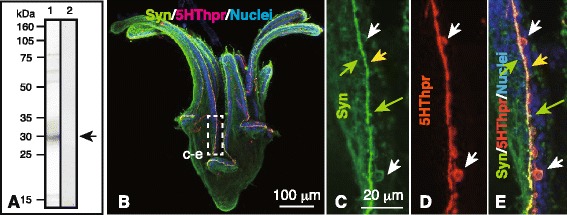
Immunochemical detection of synaptophysin (Syn) in the ciliary band-associated strand (CBAS). (**a**) Immunoblotting of the 6-arm plutei lysate was probed with rabbit anti-sea urchin-Syn pAb (*lane 1*) and detected a single band in the 30-kDa region (*arrow*). Lane 2: Rabbit pre-immune serum. (b–e) Confocal laser scanning micrographs of whole-mount immunohistochemistry of a 6-arm pluteus. (**b**) Left side view of the pluteus. The CBAS was positive for Syn (*green*) and the 5HT receptor (5HThpr; *red*). (c–e) High-magnification of the CBAS indicated by box (c–e) in (B). (**c**) Syn expression. (**d**) 5HThpr expression. (**e**) Merged image between (c) and (d) with nuclei stained with 4′,6-diamidino-2-phenylindole (*blue*). Green arrows; apical protrusions from the CBAS; white arrows, perikarya; yellow arrow, granular expression of Syn in the axon-like region of the CBAS

The Sea Urchin Genome Sequencing Project (SUGSP) has cloned a 29-kDa Sp-Syn, which is a Syn homologue of *Strongylocentrotus purpuratus* (http://sugp.caltech.edu/SpBase/search/viewAnnoGeneInfo.php?spu_id=SPU_014316). We generated an anti-Sp-Syn pAb in rabbits. The pAb bound to a single band at approximately 30 kDa (Fig. 8a, lane 1), which was similar to the predicted *M*_*r*_ of Sp-Syn. Immunospecificity of the pAbs was validated by a negative immunoreaction with rabbit pre-immune serum (Fig. 8a, lane 2). Double-stained WMIHC using pAbs against Syn and 5HThpr specifically detected these epitopes in the CBAS (Fig. 8b–e), including the apical protrusions (Fig. 8c, green arrows). Syn was associated with granular features in the axon-like region (Fig. 8c, e, yellow arrow). However, it was only weakly expressed in perikarya (Fig. 8e, white arrows). Such granular Sp-Syn expression resembles that reported in rat embryonic hippocampal neurons [26]. In contrast, anti-5HThpr pAb binding showed a smeared signal throughout the CBAS (Fig. 8d, e). Rabbit pre-immune serum did not bind to the CBAS (Fig. 7f), suggesting the immunospecificity of anti-Syn pAb. Thus, Syn may be present in the axon-like region of the CBAS.

### Lack of tubulin expression in the CBAS

The present morphological and proteomic properties of the CBAS resemble those of neurons, suggesting the presence of axonal cytoskeletons, including microtubules [27, 28]. The SUGSP detected α-tubulin (http://www.spbase.org/SpBase/search/viewAnnoGeneInfo.php?spu_id=SPU_004143) [29], acetylated α-tubulin [30], and β-tubulin [31, 32], which are the major axonal microtubule components [33, 34]. Thus, although microtubules were not detected in the CBAS by TEM (Fig. 4), the presence of these tubulin proteins was examined here by immunochemistry.

**Fig. 9:**
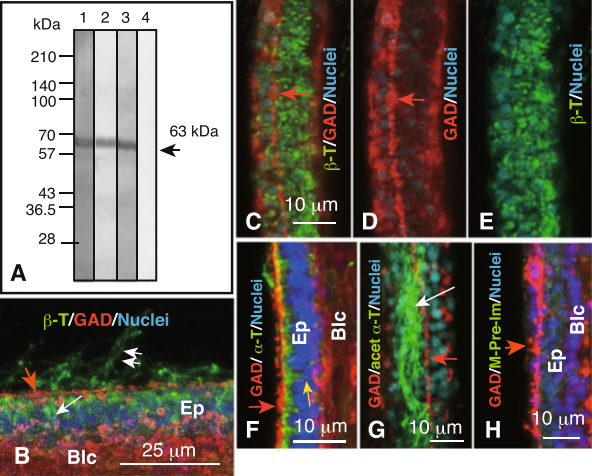
Immunohistochemistry of cytoplasmic tubulins in the ciliary band (CB)-associated strand (CBAS). **a** Immunoblotting of the 6-arm plutei lysate with mouse anti-β-tubulin monoclonal antibody (mAb) (*lane 1*), mouse anti-acetylated α-tubulin mAb (*lane 2*), mouse anti-α-tubulin mAb (*lane 3*), and mouse pre-immune serum (*lane 4*). Arrow indicates the 63-kDa region. **b**–**h** Confocal laser scanning micrographs. Nuclei were stained with 4′,6-diamidino-2-phenylindole (*blue*). **b** One-μm thick single optical longitudinal cross-section of the triple-stained CBAS. Double-arrow, cilia; red arrow, the CBAS positive to anti-GAD pAb; white arrow, cytoplasmic β-tubulin (*green*). **c**–**e** and **g**; apical surface views of the larval arms. **c** Twelve-μm thick stacked image of the CBAS (*red arrow*). **d**, **e** Color channel arranged images of (**c**). **d** Twelve-μm thick stack image of the CBAS stained with anti-GAD pAb (*red arrow*). **e** Twelve-μm thick stack image stained with anti-β-tubulin mAb (*green*). **f** Triple-stained 21-μm thick longitudinal stack image of optical cross sections of larval arm. The CBAS was stained with anti-GAD pAb (*red*). Yellow arrow, cytoplasmic α-tubulin (*green*). **g** Triple-stained 28-μm thick stack image of optical cross sections of the CBAS that was stained with anti-GAD pAb (*red, red arrow*). CB cilia were stained with mouse anti-acetylated α-tubulin (*green*). White arrow, CB cilia. **h** Fourteen-μm thick stack image of optical cross sections of mouse pre-immune serum-treated (*green*, not shown) larval arm. Blc, blastocoel; Ep, epithelium of the larval arm

Immunoblotting of 6-arm plutei lysates with mAbs against β- (Fig. 9a, lane 1), acetylated α- (Fig. 9a, lane 2), and α-tubulin (Fig. 9a, lane 3) detected binding as a single band at about 63 kDa, whereas pre-immune serum did not bind to this region (Fig. 9a, lane 4), suggesting the presence of these tubulin epitopes in plutei.

The present WMIHC detected β-tubulin in the cytoplasm immediately beneath the epithelial cell plasma membranes (Fig. 9b, arrow) and in CB cilia in optical tangential sections along the CBAS but it was not co-localized with the CBAS itself (Fig. 9b, red arrow). An overview of the apical surface did not indicate co-localization of the CBAS GAD-positive signal with β-tubulin (Fig. 9c–e). Thus, WMIHC strongly suggested the absence of β-tubulin epitope in the CBAS. However, the β-tubulin epitope was abundant in non-CBAS epithelial cells (Fig. 9e). The α-tubulin epitope was not detected in cilia or the CBAS (Fig. 9f, white arrows) but was detected again in the cytoplasm of epithelial cells (Fig. 9f). In contrast to the above two tubulins, acetylated α-tubulin was detected only in cilia (Fig. 9g, white arrow) but not in the cytoplasm of epithelial cells or the CBAS (Fig. 9g, red arrow). The mouse pre-immune serum did not exhibit positive signals in either the cilia or cytoplasm of the epithelium (Fig. 9h), which was consistent with the immunospecificity of the mAbs used to detect the positive signals.

Thus, none of the tubulins examined in the present study was detected in the CBAS, which was consistent with the absence of microtubules in the CBAS according to TEM (Fig. 4).

## Discussion

### Localization of the CBAS on the oral ectoderm side of the CB

The present WMIHC results indicate that the CBAS is localized on the oral ectoderm side of the CB that is closely associated with synaptotagmin-expressing serotonergic neural fibers in sea urchin larvae [10, 35, 36] and tornaria of the hemichordate *Ptychodera flava* [37]. In contrast, serotonergic neural fibers that extended from the apical ganglion inserted into the postoral arms but not to the other larval arms in 6-arm plutei of the present sea urchin (data not shown), and in the postoral arms they are localized on the basal surface of the CB [38]. Thus, the CBAS appears to be structurally separated from serotonergic system and is a distinct neuronal system in the larval arm epithelium.

According to the gene regulatory network (GRN) analysis, the CB is specified to the region between the oral and aboral ectoderm under regulation of transforming growth factor TGFβ signaling, and Nodal (a subset of the TGFβ superfamily) positions the oral margin of the CB in sea urchin embryos [36, 39]. Thus, Nodal signaling may also regulate CBAS position. However, further analysis of its GRN is forthcoming. The oral-aboral polarity of the CBAS was distinct in perikarya that express Epith-2. Thus, an Epith-2-related specification mechanism could exist for oral-aboral polarity of the perikaryon in the CBAS.

### Morphological and molecular properties of the CBAS

The CBAS comprises tandemly aligned approximately 60-μm long bipolar cells that extend long and thin axon-like regions from the perikaryon [7]. The CBAS was integrated into the epithelium of the larval arms by AJs along with squamous and CB cells. The epithelial cells of the sea urchin, *Lytechinus pictus*, form septate desmosome junctions between adjacent epithelial cells on the apical side during the early mesenchyme blastula stage [40]. However, septate desmosomes were rarely detected in the present study (data not shown). Such apparent differences in cell junction type in the present 6-arm plutei could be due to the different developmental stages examined. Formation of the ectodermal cell junction is initiated in *Drosophila* embryos from simple basal AJs during the early stages of “cellularization” to complex combinations of spot AJs, zonula adherens, and basal septate junctions during the stage after germ band retraction [41].

AJs are a cell-cell adhesion device that plays a vital role during epithelial morphogenesis to maintain epithelial integrity in sensory organs, such as the chicken sensory organ [42], the lateral line of zebrafish [43], and the anterior sensory organ of *Caenorhabditis elegans* [44]. However, recent studies of *Drosophila* embryos revealed that AJs mediate intercellular movement of E-cadherin and epidermal growth factor receptor-containing vesicles [45]; thus, AJs mediate cell-to-cell signaling between adjacent epithelial cells, suggesting potential cell-to-cell signal transmission between the CBAS and the CB, as will be discussed later.

The sporadic apical protrusions from the CBAS were not associated with microtubules and contained numerous 40–80 nm diameter vesicles that resembled boutons at the presynaptic terminals of presynaptic axons [46]. The present immunohistochemical detection of Syn in the axon-like region and the bouton-like protrusions of the CBAS is consistent with the present TEM observations and suggests presynaptic properties [46, 47] of the CBAS.

Thus, these morphological properties of the CBAS suggest its involvement in signal transmission activity, which will be discussed later.

### Potential sensory function of the CBAS

Larval swimming activity is solely generated by ciliary beating, which is organized into the circumoral CB in sea urchins [7, 9, 18, 35, 48]. The CB is also closely associated with several neural circuits, including serotonergic [38] and nitric oxidase synthetase (NOS)-expressing cells [35] on the basal side and GABA-ergic cells on the apical side of the larval arm epithelium [19]. NOS-expressing cells function as chemosensory cells and are involved in larval settlement and metamorphosis [35].

In the marine mollusk, *Hermissenda crassicornis*, 5HT and GABA modulate phototaxis [49]. 5HT receptors are expressed in sensory organs and transmit the light energy of solar radiation into local and systemic responses of the animal, such as in mammalian skin [50]. All receptor proteins detected in the CBAS in the present and our previous reports [7, 8, 11, 17] are involved in larval swimming, which is vital for neurotransmitter-triggered metamorphosis [1, 6]. These neurotransmitters are abundant in marine algae; 5HT in red algae, *P. yezoensis* [12, 51], GABA in red algae [5, 6], *Perna viridis*, and DA in green algae, *Ulvaria obscura* [13, 14]. The presence of receptor proteins for these neurotransmitters in the CBAS supports the hypothesis that the CBAS functions as a sensory organ for these neurotransmitters released from environmental algae to activate the larval settlement program and or seek habitat where larvae can hide from predators.

The presence of Syn in the CBAS is another observation supporting the sensory function. Neural signal transmission-associated Syn is expressed in sensory cells, such as the auditory organ of chicks [52] and humans [53], taste bud cells in rats [54], rhodopsin-positive cells of rats [55], the human retina [56], and the lateral line of the blind cavefish larva [57] and zebrafish [58].

Larval settlement and metamorphosis are also induced by histamine in the sea urchin *Holopneustes purpurascens* [59] and *S. purpuratus* [60]. Histamine is contained in several algae, such as red algae *Delisea pulchra* and coralline turf algae [59]. Histamine receptor 1 (suH1R) activates the nitric oxide pathway in *S. purpuratus* [61] and is expressed associated with the plasma membrane of large area of ectodermal cells [60]. Regarding NOS-expressing cell localization near the CB and its involvement in larval settlement and metamorphosis [35], characterization of functional and structural means between the histaminergic system and the CBAS will further promote functional understanding of the CBAS.

### Cell surface properties of the CBAS

Axons and perikarya of serotonergic neurons in sea urchin embryos and larvae that originate from the animal ectoderm of the embryo approximately at the prism stage express the epithelial cell surface-specific Epith-2 protein on the entire cell surface, including axons [38], whereas Epith-2 is expressed only on the surface of the perikarya in the CBAS. Because (1) Epith-2 and its sister protein Epith-1 are expressed exclusively on the lateral surface of epithelial cells [20, 21], (2) GADCs formed by delamination from the embryonic ectoderm via the EMT acquire a mesenchymal cell surface property by losing Epith-2 from the entire cell surface [19, 22], and (3) as the present results indicated the CBAS possessed similar cytoplasmic property to blastocoelar GADCs by co-expressing GAD and 5HThpr, the CBAS may be derived from blastocoelar GADCs through the mesenchymal-to-epithelial transition (MET). Although further studies ought to be done, it could be interpreted that exclusive expression of Epith-2 on the perikarya observed in the present study suggests “restoration” of the original heterogeneous epithelial cell surface property during or after formation. The MET has been reported to occur during various developmental morphogenetic processes, such as in endothelium and dermal formation from neural crest cells [62], wound healing, and metanephric kidney development [63]. Cell surface properties are modified during several MET processes, such as wound healing [63] and early differentiation of hepatic stem cells in mice [64]. Further studies of the MET during CBAS formation may shed new light on the developmental mechanism of the CBAS.

### Absence of microtubules and tubulins in the CBAS

The structural unit of the CBAS is a bipolar GADC with a bulbous perikaryon and thin, long-stretched axon-like regions [7]. The diameter of the axon-like region is 1–2 μm, which is comparable to that of serotonergic axons of early sea urchin plutei [38]. The axons of other marine invertebrates, such as crayfish, lobster [65], annelids, *Neanthes arenaceodentata* [66], and *Platynereis* larvae [67], have been reported to contain microtubules. However, microtubules have not been observed in the serotonergic axons of sea urchin larvae. Because microtubules are vital to maintain axonal structure and transport [68, 69], they are considered structural and molecular markers of axons/neurons. The present observation of the absence of microtubules in the CBAS may reflect instability of the microtubules [70]. Consistent with this interpretation, the genes and proteins of α-, β-, acetylated α- and βIII-tubulin have been reported in sea urchins [29–32, 71], and microtubules have been detected in embryonic ectodermal cells of the sea urchin, *L. pictus* by TEM [40], in the preoral epithelium of *H. pulcherrimus* [72], and in larval epithelial cells in the present study (Fig. 6). Thus, instability of microtubules could be characteristic of axons and the CBAS of sea urchin larvae.

However, further progress on gene annotation in the SUGSP may depict such orthologous genes and proteins based on a report of a neural α-tubulin in *Paracentrotus lividus* [73], and, thus, they will be immunochemically detectable in a future study.

The characteristic apical protrusions that extended from the CBAS toward the CB (Figs. 3 and 5) resembled synaptic boutons [74]. Although such structures are suggestive of a neuronal signal transmitting mechanism of the CBAS, further characterization is needed using simultaneous photobleaching and imaging techniques [74]. Identifying the targets of the apical protrusions will also shed light on the physiological role of the CBAS.

## Methods

Sea urchins (*H. pulcherrimus* A. Agassiz) were collected near the Research Center for Marine Biology, Tohoku University, Japan or the Marine and Coastal Research Center, Ochanomizu University, Japan. Gametes were obtained by intracoelomic injection of 0.5 M KCl. Eggs were inseminated and incubated in filtered sea water (FSW) on a gyratory shaker or stirred gently with a propeller in plastic containers in an incubator at 15 or 18 °C until the appropriate developmental stages were reached. Larvae were fed *Chaetoceros calcitrans* (Nisshin Marine Tech. Ltd., Yokohama, Japan) beginning 4 days after fertilization.

### Raising antibodies

The anti-sea urchin Syn pAb was raised in rabbits based on the *S. purpuratus* Syn peptide sequence (SPU_014316.3a; Sp-Syn). The epitopic amino acid sequence, ^241^KETTWFKQRMENKAGGAA^258^, was chosen based on a proteomics analysis at NPS@: Network Protein Sequence Analysis ([75]: https://npsa-prabi.ibcp.fr/cgi-bin/npsa_automat.pl?page=npsa_pcprof.html). The peptide was near the C-terminus of the protein, predicted to extend into the cytoplasm, and was tagged with keyhole limpet hemocyanin (KLH) at the N-terminus through a cysteine residue. The KLH-tagged synthetic peptide was inoculated into a rabbit four times in 56 days. A Basic Local Alignment Search Tool (BLAST) analysis by SpBase (http://www.spbase.org/SpBase/wwwblast/blast.php) and the National Center for Biotechnology Information (NCBI; http://blast.ncbi.nlm.nih.gov/Blast.cgi?PROGRAM=blastp&PAGE_TYPE=BlastSearch&LINK_LOC=blasthome) did not detect any antigen peptide sequence similar to the present antigen peptide in any other sea urchin proteins registered in the protein database; thus, this sequence was considered unique to Sp-Syn. The antiserum was used as anti-Sp-Syn pAb for the immunochemical analyses.

The anti-sea urchin ECPN polyclonal pAb was raised in a rabbit based on *H. pulcherrimus* ECPN (Hp-ECPN; AB458218) as described for mice using the same amino acid sequence [8].

### Immunoblotting

Immunospecificity of the mAbs raised against chicken α-tubulin (clone N356; Amersham Pharmacia Biotech, Piscataway, NJ. USA), sea urchin (*S. purpuratus*) acetylated α-tubulin (clone 6-11B-1, Sigma, St. Louis, MO. USA), and sea urchin (*S. purpuratus*) β-tubulin (clone 2-28-33, Sigma) were diluted 1:1000 in Tris-buffered saline with 1 % Tween-20 (v/v) (TBST). Rabbit anti-Sp-Syn pAb and anti-Hp-ECPN pAbs were diluted 1:1000 and 1:750 respectively in TBST. Rabbit pre-immune serum and mouse pre-immune serum (diluted 1:1000 in TBST) were applied as negative immunoblotting controls in lyophilized 4-arm and 6-arm plutei. The samples were diluted in 0.1 M sample buffer with 2-mercaptoethanol and separated by 10 % uniform sodium dodecyl sulfate-polyacrylamide gel electrophoresis under reducing conditions, blotted to a polyvinylidene difluoride membrane, blocked with 5 % (w/v) skim milk diluted in TBST, and incubated with the above described pAbs or mAbs for 2 h at ambient temperature (AT). The primary antibodies were detected by incubation with alkaline phosphatase (AP)-tagged anti-mouse or rabbit IgG pAbs (Sigma) diluted 1:15,000 in TBST for 2 h at AT and visualized with the chromogenic reagents nitro-blue tetrazolium and 5-bromo-4-chloro-3′-indolyphosphate (Promega, Madison, WI, USA) diluted in AP buffer (pH 9.5) according to the manufacturer’s instructions.

### Whole-mount immunohistochemistry

Embryos and larvae that had reached the appropriate developmental stage were fixed in 4 % paraformaldehyde (diluted in FSW) for 15–20 min at AT, dehydrated, and stored at 4 °C until use. Then, the samples were hydrated in a series of decreasing ethanol concentrations (to 30 %) and transferred to 0.1 M phosphate-buffered saline (pH 7) with 1 % Tween-20 (PBST).

The samples were blocked with 1 % bovine serum albumin in PBST for 1 h and exposed to rabbit anti-rat GAD_65/67_ pAb (Enzo Life Sciences International, Plymouth Meeting, PA, USA; diluted 1:500 in PBST), mouse anti-5HThpr pAb [*H. pulcherrimus* serotonin receptor [8, 9, 19, 22] diluted 1:200 in PBST], mouse Epith-2 mAb [anti-epithelial cell surface-specific mAb [20, 21] diluted 1:100 in PBST], rabbit anti-sea urchin DRD1 pAb ([10]: diluted 1:50 in PBST), mouse anti-sea urchin β-tubulin mAb (Sigma, diluted 1:500 in PBST), mouse anti-chicken α-tubulin mAb (Amersham Pharmacia, diluted 1:500 in PBST), mouse anti-sea urchin acetylated α-tubulin mAb (Sigma, diluted 1:500 in PBST), rabbit anti-Sp-Syn pAb (diluted 1:300 in PBST), or rabbit anti-Hp-ECPN pAb (diluted 1:500 in PBST) for 2 days at 4 °C. The present rabbit anti-rat GAD_65/67_ pAb was raised against the synthetic peptide (^572^DFLIEEIERLGQDL^585^) from rat GAD_65/67_, which is quite similar to GAD (^597^DFMLDEIERLGKPL^605^) of sea urchin. The immunospecificity of rabbit anti-rat GAD_65/67_ pAb to sea urchin GAD was confirmed in our previous study [19]. Rabbit pre-immune serum (diluted 1:500 in PBST) and mouse pre-immune serum (diluted 1:500 in PBST) were used as negative controls.

The primary Abs were visualized with Alexa Fluor 488- or 594-tagged anti-rabbit or -mouse IgG (H + L) Abs (diluted 1:500–750 in PBST; Invitrogen, Eugene, OR, USA). The samples were counterstained with 1 μg/ml DAPI for 5 min at AT and examined using a Leica TCS SP8 confocal laser scanning microscope (CLSM; Leica Microsystems). Images were analyzed with ImageJ (NIH, Bethesda, MD, USA) and Adobe Photoshop CS2 software (ver. 9.02, Adobe Systems Inc., La Jolla, CA, USA), and presented with projection images. The GAD/Epith-2/DAPI triple-stained WMIHC samples were further examined under a CLSM with HSR software (HSR image; Leica Microsystems).

### Three-dimensional construction of CLSM images

The images were 3D reconstructed using the 3D Visualization and Analysis Software (Amira ver. 6.0.0; FEI Visualization Sciences Group) to clarify the spatial relationship of the images obtained by WMIHC. DAPI-stained images were integrated into the images of two channels (red and green) after semi-transparent treatment. The 3D topographic model of the CBAS was reconstructed with Amira and HSR and analyzed using ImageJ animation (NIH).

### Transmission electron microscopy

The larvae were fixed in a mixture of 2.3 % monomeric glutaraldehyde (Electron Microscope Sciences, Hatfield, PA, USA), 0.2 % dimeric glutaraldehyde [absorbance value > 0.250 at 235 nm against distilled water (DW), as a 0.1 % aqueous solution], 0.36 M sucrose, 0.08 M piperazine-N,N’-bis(ethanesulfonic acid), and 1,4-piperazinediethanesulfonic acid buffer (pH 7.4) for 1 h at AT and post-fixed in 1 % OsO_4_ in DW for 1 h. The dehydrated specimens were embedded in Spurr’s resin (Electron Microscope Sciences), and all sections were made with a Leica EM UC7 ultramicrotome (Leica Microsystems GmbH, Vienna, Austria). The orientation of the section was examined under a stereomicroscope. Thick sections were stained with 1 % toluidine blue. Thin sections (110 nm) were stained with 3 % lead citrate and 0.5 % uranium acetate, respectively and examined under a JEM1400 Plus TEM (JEOL Ltd., Tokyo, Japan) at 80 kV. Image size was 3296 × 2472 pixels.

### Three-dimensional construction of TEM images

TEM 3D topographic reconstruction of the CBAS postoral arm was performed by stacking nine 110-nm thick semi-serial sections using Amira image software (ver. 6.0.0). These thin sections encompassed 32-μm long sections of an arm, and each thin section was taken every 4 μm from the distal to the proximal region of the arm using a Leica EM UC7 ultramicrotome. These thin sections were digitally captured and the images were processed and oriented in Adobe Photoshop CS5. The images were imported into Amira vr.6.0.0 and aligned into a single stack. To compensate for the number of serial thin sections, 405 virtual images between the thin sections were calculated using “Interpolate Labels”. The final tomographic model was produced by post-processing including surface rendering and smoothing, and analyzed using ImageJ animation (NIH).

## Conclusions

The unique morphology of the CBAS in the sea urchin larva epithelium had not been reported. The CBAS expresses a remarkable number of receptors to environmental stimuli and proteins that are probably involved in signal transmission to the CB. The properties of the CBAS explain previous reports that larval swimming is triggered by environmental stimuli and suggest crosstalk among receptors and potential plural sensory functions of the CBAS.

## Abbreviations

3D, three-dimension; 5HT, serotonin; 5HThpr, serotonin receptor of *Hemicentrotus pulcherrimu*s; AJ, adherens junction; ala, anterolateral arm; AP, alkaline phosphatase; AT, ambient temperature; CB, Ciliary band; CBAS, ciliary band-associated strand; CLSM, confocal laser scanning microscope; DA, dopamine; DAPI, 4′,6-diamidino-2-phenylindole; DRD1, dopamine receptor D1; ECPN, encephalopsin; EMT, epithelial-to-mesenchymal transition; FSW, filtered seawater; GABA, γ-aminobutyric acid; GAD, glutamate decarboxylase; GADC, glutamate decarboxylase-expressing cell; GRN, gene regulatory network; HSR, Hybrid Super Resolution; IgG, immunoglobulin G; mAb, monoclonal antibody; NOS, nitric oxidase synthetase; pAb, polyclonal antibody; PBST, phosphate-buffered saline with 1 % Tween-20; pda, posterodorsal arm; poa, postoral arm; Sp, *Strongylocentrotus purpuratus*; SUGSP, Sea Urchin Genome Sequencing Project; Syn, synaptophysin; TBST, Tris-buffered saline with 1 % Tween-20; TEM, transmission electron microscopy; TGFβ, transforming growth factor β; WMIHC, whole-mount immunohistochemistry; MET, mesenchymal-to-epithelial transition
